# Implementation of an intraoperative electron radiotherapy in vivo dosimetry program

**DOI:** 10.1186/s13014-016-0621-y

**Published:** 2016-03-15

**Authors:** Juan López-Tarjuelo, Virginia Morillo-Macías, Ana Bouché-Babiloni, Enrique Boldó-Roda, Rafael Lozoya-Albacar, Carlos Ferrer-Albiach

**Affiliations:** Servicio de Radiofísica y Protección Radiológica, Consorcio Hospitalario Provincial de Castellón, Avda. Dr. Clará, nº 19, Castellón de la Plana, 12004 Castellón Spain; Servicio de Oncología Radioterápica, Consorcio Hospitalario Provincial de Castellón, Castellón de la Plana, Spain; Unitat predepartamental de Medicina, Facultat de Ciències de la Salut, Universitat Jaume I, Avda. Vicent Sos Baynat, s/n, Castellón de la Plana, 12071 Castellón Spain; Unidad de Cirugía Oncológica, Servicio de Cirugía, Consorcio Hospitalario Provincial de Castellón, Castellón de la Plana, Spain; Departamento de Medicina y Cirugía, Facultad de Ciencias de la Salud, Universidad Cardenal Herrera-CEU, C/ Grecia 31, Castellón de la Plana, 12006 Castellón Spain

**Keywords:** Intraoperative radiotherapy, In vivo dosimetry, MOSFETs, Radiochromic films, Oncological surgery

## Abstract

**Background:**

Intraoperative electron radiotherapy (IOERT) is a highly selective radiotherapy technique which aims to treat restricted anatomic volumes during oncological surgery and is now the subject of intense re-evaluation. In vivo dosimetry has been recommended for IOERT and has been identified as a risk-reduction intervention in the context of an IOERT risk analysis. Despite reports of fruitful experiences, information about in vivo dosimetry in intraoperative radiotherapy is somewhat scarce. Therefore, the aim of this paper is to report our experience in developing a program of in vivo dosimetry for IOERT, from both multidisciplinary and practical approaches, in a consistent patient series. We also report several current weaknesses.

**Methods:**

Reinforced TN-502RDM-H mobile metal oxide semiconductor field effect transistors (MOSFETs) and Gafchromic MD-55-2 films were used as a redundant in vivo treatment verification system with an Elekta Precise fixed linear accelerator for calibrations and treatments. In vivo dosimetry was performed in 45 patients in cases involving primary tumors or relapses. The most frequent primary tumors were breast (37 %) and colorectal (29 %), and local recurrences among relapses was 83 %. We made 50 attempts to measure with MOSFETs and 48 attempts to measure with films in the treatment zones. The surgical team placed both detectors with supervision from the radiation oncologist and following their instructions.

**Results:**

The program was considered an overall success by the different professionals involved. The absorbed doses measured with MOSFETs and films were 93.8 ± 6.7 % and 97.9 ± 9.0 % (mean ± *SD*) respectively using a scale in which 90 % is the prescribed dose and 100 % is the maximum absorbed dose delivered by the beam. However, in 10 % of cases we experienced dosimetric problems due to detector misalignment, a situation which might be avoided with additional checks. The useful MOSFET lifetime length and the film sterilization procedure should also be controlled.

**Conclusions:**

It is feasible to establish an in vivo dosimetry program for a wide set of locations treated with IOERT using a multidisciplinary approach according to the skills of the professionals present and the detectors used; oncological surgeons’ commitment is key to success in this context. Films are more unstable and show higher uncertainty than MOSFETs but are cheaper and are useful and convenient if real-time treatment monitoring is not necessary.

## Background

Intraoperative electron radiotherapy (IOERT) is a highly selective radiotherapy technique used to treat restricted anatomical volumes during oncological surgery. Single-fraction irradiation with a high absorbed-dose is delivered by means of an electron beam, after direct visual examination of the tumor bed or non-resectable tumor [1]. It allows the biological effect of ionizing radiation to be maximized while minimizing or avoiding exposure to adjacent at-risk organs. This technique is potentially beneficial because it shortens the treatment time required compared to conventional fractionation, and it does not interfere with the administration of systemic therapy [2].

IOERT is currently the subject of intense review [3–5]. Therefore, in this context it is pertinent to also re-assess in vivo dosimetry, which is generally recommended for end-to-end tests [6] and to assess treatment verifications [7], particularly for IOERT [8] where it has been identified as a risk-reduction intervention in the context of an IOERT risk analysis [9].

Fruitful experiences based either on metal oxide semiconductor field effect transistors (MOSFETs) [10–12], radiochromic films [13, 14], or both [15] have been reported. However, information about in vivo dosimetry in intraoperative radiotherapy is somewhat scarce. Therefore, the aim of this paper is to report our experience in developing a program of in vivo dosimetry for IOERT, from both multidisciplinary and practical approaches, in a consistent patient series.

## Methods

### Dosimetric equipment

Reinforced TN-502RDM-H mobile MOSFETs (Best Medical Canada Ltd., Ontario, Canada) and Gafchromic MD-55-2 film (International Specialty Products, NJ, USA) were used as a redundant in vivo treatment verification system. The absorbed doses used in the calibration procedure were accurately measured by a medical physicist using routine dosimetric equipment following the International Atomic Energy Agency’s TRS-398 protocol [16] immediately before irradiating the dosimeters. MOSFETs were read by selecting the standard sensitivity bias setting [17]. A third-order polynomial relationship between pixel value, read in the red channel, and absorbed dose was used to calibrate the film. Films, cut into 1.5 cm × 1.5 cm pieces, were read by an Epson perfection V700 Photo flatbed digitizer (Seiko Epson Corporation, Nagano, Japan). An Elekta Precise fixed linear accelerator (linac; Elekta AB, Stockholm, Sweden) was used both for calibrations and treatments. The linac is equipped with dedicated trays, capable of allocating cylindrical applicators of 3, 4, 5, 6, 7, 8, 9, 10, 12, and 15 cm in diameter and 5 mm in thickness, made of polycarbonate (MCP Iberia S.A., Madrid, Spain). All of them have interchangeable distal ends with no bevel, a 30°-bevel, and a 45°-bevel which result in a nominal source-to-applicator-end distance of 135 cm. The table used for treatments was a SU-14 movable operating table equipped with 150 mm diameter wheels with one directional wheel (Famed Żywiec Sp. z o. o., Żywiec, Poland).

### Patients and measurements

**Fig. 1:**
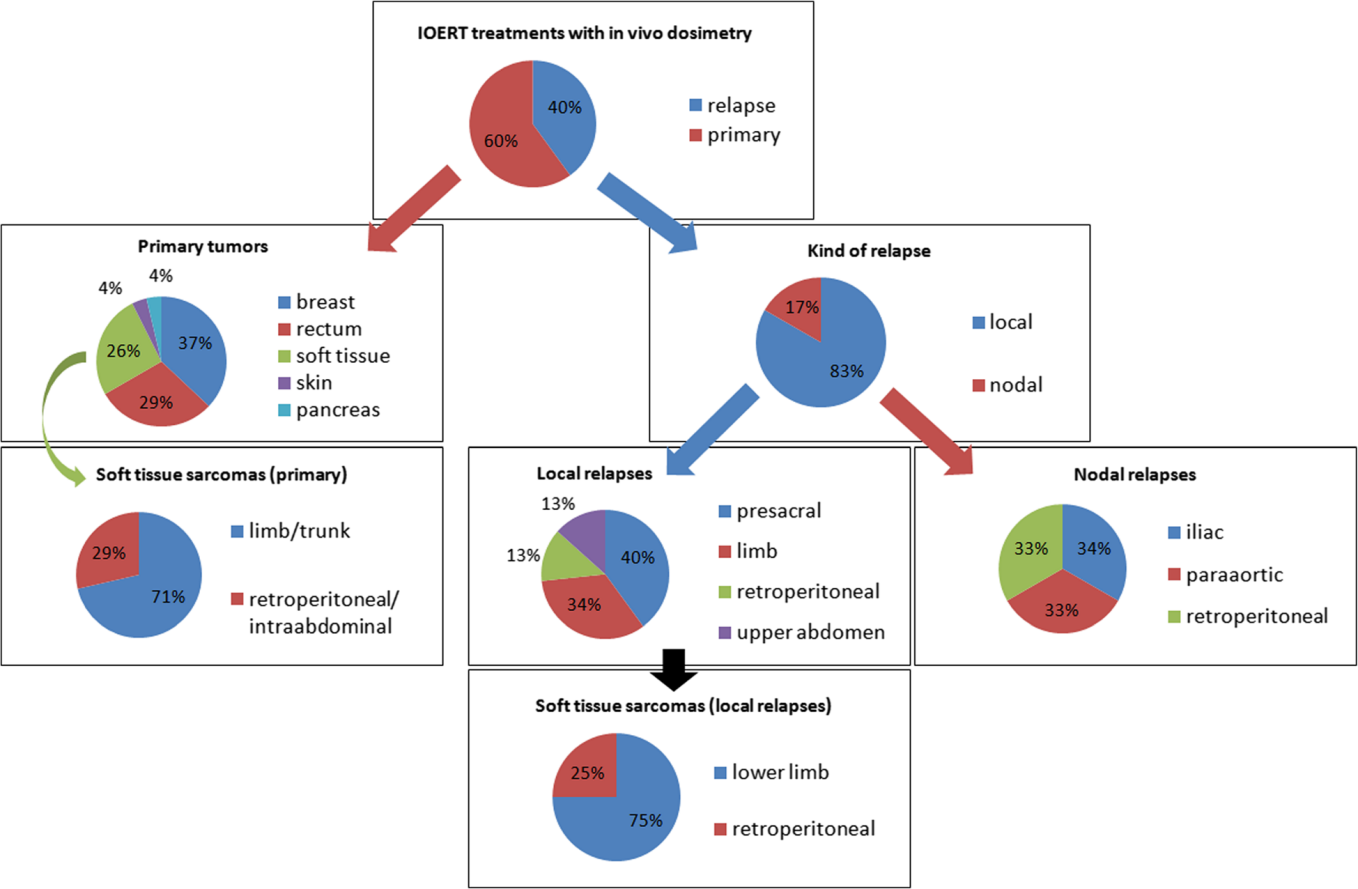
Distribution of neoplasms according to treatment intention and type

We performed in vivo dosimetry in 45 patients from March 2011 to January 2014 in cases involving primary tumors (60 %) or relapses (40 %). Detectors were placed in a variety of sites which are classified as depicted in Fig. 1. The most frequently treated primary tumors were breast (37 %) and colorectal (29 %), and the predominance of local recurrences among relapses was 83 %. Fifty attempts to measure with MOSFETs and 48 attempts to measure with films were made in the treatment zones. The number of attempts was greater than the number of patients because radiation oncologists documented other measurements in the tumor bed for a few initial cases. In addition to this, an extra film was not available for the in vivo procedure in a couple of measurements of this kind. However, for the purpose of completeness, below we analyze all the valid readouts obtained. In 35 cases we were able to simultaneously measure with a MOSFET and a film. These measurements were carefully examined along with their setup to identify any anomalous events. Afterward, we assessed the outliers of the remaining data, which were defined as measurements exceeding three standard deviations (*SD*s) of the sample means, and were considered not to be representative of the dose delivered to the tumor bed.

### Surgical aspects

The surgical team placed the MOSFETs and radiochromic films with supervision by the radiation oncologist and following their instructions. The standard placement was: one MOSFET and one radiochromic film at the center and another optional MOSFET at the periphery of the radiation field but out of the penumbral zone. As the team gained confidence the detectors were restricted to one MOSFET per patient because of economic factors. MOSFETs were always fixed to the radiation field by two stitches in order to avoid it moving during the procedure. When the surgical field was not horizontal (i.e. in the vertical presacral field), the radiochromic film was also fixed with stitches.

### Ethical statement

This research was approved by our institution’s ethics committee in accordance with the ethical standards laid down in the 1964 Declaration of Helsinki and its later amendments. Written informed consent for this procedure was obtained from every patient.

## Results

The program was considered an overall success as evaluated by the different professionals involved. The time between exiting the operating room (OR) and administration of the radiation treatment was around 40 min, which differed slightly according to the tumor location. The irradiation parameters used and dosimetric results obtained are shown in Table 1.

**Table 1:** Distribution of dosimetric parameters, f stands for their relative frequency

Applicator diameter (cm)	f	Energy (MeV)	f	Bevel angle (°)	f	Prescribed dose (Gy)	f
4	4 %	4	6 %	0	56 %	5	6 %
5	2 %	6	17 %	30	33 %	9	8 %
6	35 %	9	52 %	45	10 %	10	8 %
7	23 %	12	19 %			12	4 %
8	13 %	15	6 %			12.5	33 %
9	13 %					15	17 %
10	6 %					17	4 %
12	4 %					17.5	6 %
						21	13 %

**Fig. 2:**
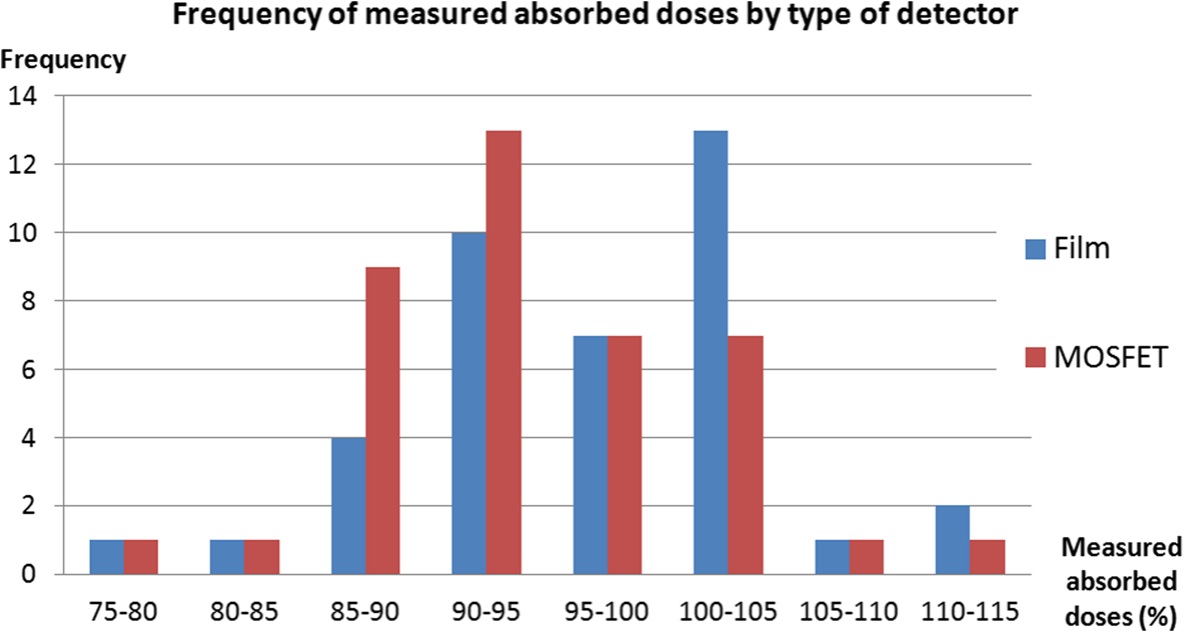
Histograms of measured absorbed doses classified by the type of detector

Of the 50 attempts to measure with a MOSFET, in 5 cases the detector could not be correctly attached to the tumor bed meaning that movements during the treatment setup pushed it towards or beyond the field limit, therefore causing low dose measurement. This happened specifically in 3 out of the 5 cases in which we placed the optional MOSFET. In the other 45 cases the detector was able to measure the absorbed dose delivered to the tumor bed. However, 3 of these 45 measurements were affected by backscattering caused by the lead protector (which is required in some cases [18]) placed next to the detector, resulting in measurements that were higher than expected in the region (in 1 case out of the 5 optional MOSFETs), and 2 recorded an anomalous low reading (less than three *SD*s from the sample mean). The descriptive statistics of the remaining 40 measurements, which were assessed as valid, are presented in Table 2 and their distribution is depicted in Fig. 2. The only valid measurement taken by an optional MOSFET differed by −6.4 % compared to the absorbed dose from the MOSFET placed in the center of the tumor bed.

**Table 2:** Descriptive statistics of the valid measurements: 40 measurements taken with MOSFETs and 42 measurements taken with films

% Absorbed dose	MOSFET	Film
Minimum	78.0	72.0
1^st^ quartile	90.0	92.7
Median	92.8	98.1
2^nd^ quartile	97.9	103.1
Maximum	111.6	123.4
Mean	93.8	97.9
Standard deviation	6.7	9.0

With regard to films, it is worth mentioning that in two cases the detector was severely damaged at an unknown point during the procedure and a readout could not be obtained. With respect to the 46 attempts to measure with a valid film, in four cases the detector could not be correctly attached to the tumor bed, and movements during treatment setup pushed it out of the field or lifted it so that the film was incorrectly irradiated at an angle very far from perpendicular. The descriptive statistics of the remaining 42 measurements, which were assessed as valid, are also presented in Table 2. Their distribution is shown in Fig. 2.

After discarding anomalous data affected by the factors mentioned above, a total of 30 paired measurements from the tumor bed were obtained; their descriptive statistics are represented in Table 3.

**Table 3:** Descriptive statistics of the paired measurements

% Absorbed dose	MOSFET	Film
Minimum	78.0	72.0
1^st^ quartile	90.2	92.4
Median	92.5	97.3
2^nd^ quartile	97.6	103.0
Maximum	111.6	123.4
Mean	93.9	97.9
Standard deviation	6.8	9.7

Thirty pairs of values for which the prescribed dose was 90 % and the maximum absorbed dose delivered was 100 %

## Discussion

### Calibration issues

As suggested by Agostinelli et al. [12], we calibrated every MOSFET, and based on the energy used. Our calibration factors (CFs) ranged from 97.1 to 104.5 mV/Gy, with a mean of 101.2 mV/Gy. They reported a similar result with a mean CF of 105 mV/Gy [12]. The mean reproducibility of our CFs in terms of the mean relative *SD* was 1.5 % which is very close to the 2 % obtained also by Agostinelli et al. [12] and Bloemen-van Gurp et al. [17]. The CF variability leads us to recommend performing a different MOSFET calibration for every energy used. Nonetheless, the calibration factors obtained under specific circumstances can be very similar, which has motivated the use of only some energies [19]. In contrast, the film readout is independent of the therapy beam energies [20]. A session which measures both the dose delivered by the linac and the calibration of one of the detectors spends approximately 1 h of linac time and so it is not as time consuming. In the case of films, the user should also consider both hardware and software needs. As a minimum a flatbed digitizer and image processing software capable of splitting the signals from different channels and evaluating statistical data inside defined regions of interest [21] should be used. Moreover, the direct use of net red pixel values to fit the film calibration curve (*r*^*2*^ > 0.999) is as satisfactory as the use of optical densities [22, 23].

### Surgical procedure and detector handling

Despite the surgical team’s effort to obtain proper and reliable sensor placement, dosimetry failed in around 10 % of cases, presumably because of unintended detector movements and misalignment. We experienced the latter problem when we tried to measure using two MOSFETs at the same time, as reported in the results section. The difficulty of inserting more than one MOSFET has also been identified elsewhere [24]. In addition, it has been pointed out that MOSFETs are presumably more difficult for the surgeon to initially handle because they have to be inserted with sterile components and attached onto the surgical bed [15]. Moreover, the compression placed on the MOSFET by the applicator can also move it [10]. However, we did not find any relationship between particular surgeons or difficulty of access to the surgical bed in these affected cases, and so we recommend that double-checking the detector placement before irradiation may reduce this problem in some cases.

Given the above circumstances, after treating the first 23 patients, measurements were performed only in the center of the tumor bed, when the team had gained confidence with respect to measurements in that zone, and thus we considered the learning curve to be surpassed. This decision implied savings in the cost of MOSFETs and catheters.

Backscattering might be addressed by adding bolus cut-outs to the lead protectors, however, the surgical team should evaluate the feasibility of this solution (which involves intensive handling of external pieces on the tumor bed) before it is routinely implemented.

In one case the MOSFET was depleted during the treatment and therefore subsequent readout was not possible. To avoid this pitfall, we recommended that the dosimeter gate voltage be read before the treatment is administered. We presume that film damage occurred during sterilization, indicating that the stability of the sterilization procedure should also be checked.

The detectors were attached in the OR before patient transportation and while the linac bunker was being prepared for the irradiation. This is why in vivo dosimetry only took 5 min more, representing the time spent verifying the detector placement just before patient treatment.

### The impact of using a fixed linear accelerator

When IOERT was first introduced into medicine it was performed by using conventional linacs in the radiotherapy treatment vaults [1]. However, in the 1990s, dedicated mobile linacs, characterized by output rates of 2 to 12 cGy per pulse, were developed and introduced [4]. The high dose-per-pulse reduces the irradiation time during the surgery because 10 Gy are typically delivered in less than 1 min [24].

Non-exclusive fixed linacs offer several advantages over the mobile ones as they allow verification of proper alignment between the treatment field and the collimator: a so-called *pseudo*-*guided image* [25]. In addition, they supply a wide range of energies that provide better coverage in response to the thickness of the target volume. The stability of their output is also better, with day-to-day variations equal to or less than 0.4 %, i.e. 1 *SD* [26]. This is one order of magnitude less than the output stability of mobile linacs [12]. Nevertheless, fixed linacs require more infrastructure, i.e. a more complex organizational system which is specifically designed for development of the procedure. They also require high-level cooperation between staff and a larger multidisciplinary team. This is why it is advisable to design detailed protocols conforming to clinical indications, as well planning the whole process in detail.

Despite these disparities between mobile and fixed linacs, in vivo dosimetry seems to perform equally well for both kinds of treatment machines [10, 11, 13, 15]. In contrast, using a mobile linac does not ensure that low variability is achieved with respect to in vivo dosimetry results, as discussed below [12].

### Dosimetry

The central values of all the distributions presented laid inside the range limited by the prescribed dose to the tumor bed in our institution (90 %) and the maximum absorbed dose (100 %). However, the spread of our measurements were wider than those reported in the literature, which ranged from 17.6 % [11] to 21.2 % [27]. This may have been influenced by the wide set of tumor locations treated, each with different degrees of complexity. Besides, our series of cases included 40 % relapses, which may involve areas already irradiated with conformal radiotherapy and/or affected by a prior surgery. These interventions favor the appearance of fibrosis [28, 29] which retracts tissues and may make dosimeter placement difficult. In the case of MOSFETs, if the maximum and minimum values are omitted, the resulting range is 23.8 % which is comparable to the results mentioned above. In this context, the work by Agostinelli et al. [12] illustrates how the range of differences between the MOSFET dose and the expected dose can be as much as 43 % even when using a single mobile machine to treat only breast tumor beds, and describes how they had to develop normalization tools to better correlate these doses. Furthermore, the need for increased precision has been reported elsewhere [30], although future imaging tools which are still in research and development may improve this issue [31].

Film measurements were more wide-ranging and tended to diverge from MOSFET measurements over time. In fact both samples were shifted to a near-significant degree (*p* = 0.070) which was presumably caused by long-term film instability. In addition, radiochromic film exhibited a greater uncertainty caused by a higher intrinsic response variation compared to MOSFET readout reproducibility. These factors do not impede dosimetry but users should be aware that they should control film response in a more intensive way than for MOSFET measurements.

### Economic factors

With regard to consumable costs, we spent over $135 per patient with MOSFET dosimetry and $2 per patient with film dosimetry (according to our institution country’s prices in December 2014). The calibration process can be easily allocated within the linac quality control sessions and so does not directly incur additional costs. The MOSFET reading equipment is more expensive than the hardware required to read films, but it can be also used to perform in vivo dosimetry for other radiotherapy techniques. The cost of a flatbed scanner is incorporated into the departmental costs as it is also used to scan documents in the department, and the sterilization costs form part of the general sterilization activities in the hospital. Finally, free access image analysis software like ImageJ (National Institutes of Health, MD, USA) is available on the internet. Unfortunately, to the knowledge of the authors, there are no other reports on these costs available. This information would also help new users to decide on the best dosimetric materials to use.

## Conclusions

It is feasible to establish an in vivo dosimetry program for a wide set of locations treated with IOERT. This program should present a multidisciplinary approach according to the skills of the professionals implementing it and with regard to the detectors in use. The commitment of oncological surgeons is key to its success because they are the professionals who are authorized to intervene in patient tumor beds and they are largely responsible for detector positioning. Films are more unstable and suffer from a higher uncertainty than MOSFETs, but they are also useful and convenient if treatment monitoring in real-time is not required.
